# Structure of fructose bisphosphate aldolase from *Encephalitozoon cuniculi*

**DOI:** 10.1107/S1744309111021841

**Published:** 2011-08-13

**Authors:** Anna Gardberg, Banumathi Sankaran, Doug Davies, Janhavi Bhandari, Bart Staker, Lance Stewart

**Affiliations:** a Emerald BioStructures, 7869 NE Day Road West, Bainbridge Island, WA 98110, USA; b Advanced Light Source, USA; c Department of Allergy and Infectious Diseases, School of Medicine, University of Washington, USA

**Keywords:** SSGCID, aldolases, substrate binding, fructose 1,6-bisphosphate, fructose bisphosphate aldolose

## Abstract

Fructose bisphosphate aldolose (FBPA) enzymes have been found in a broad range of eukaryotic and prokaryotic organisms. FBPA catalyses the cleavage of fructose 1,6-bisphosphate into glyceraldehyde 3-phosphate and dihydroxy­acetone phosphate. The SSGCID has reported several FBPA structures from pathogenic sources. Bioinformatic analysis of the genome of the eukaryotic microsporidian parasite *Encephalitozoon cuniculi* revealed an FBPA homolog. The structures of this enzyme in the presence of the native substrate FBP and also with the partial substrate analog phosphate are reported. The purified enzyme crystallized in 90 m*M* Bis-Tris propane pH 6.5, 18% PEG 3350, 18 m*M* NaKHPO_4_, 10 m*M* urea for the phosphate-bound form and 100 m*M* Bis-Tris propane pH 6.5, 20% PEG 3350, 20 m*M* fructose 1,6-­bisphosphate for the FBP-bound form. In both cases protein was present at 25 mg ml^−1^ and the sitting-drop vapour-diffusion method was used. For the FBP-bound form, a data set to 2.37 Å resolution was collected from a single crystal at 100 K. The crystal belonged to the orthorhombic space group *C*222_1_, with unit-cell parameters *a* = 121.46, *b* = 135.82, *c* = 61.54 Å. The structure was refined to a final free *R* factor of 20.8%. For the phosphate-bound form, a data set was collected to 2.00 Å resolution. The space group was also *C*222_1_ and the unit-cell parameters were *a* = 121.96, *b* = 137.61, *c* = 62.23 Å. The structure shares the typical barrel tertiary structure reported for previous FBPA structures and exhibits the same Schiff base in the active site. The quaternary structure is dimeric. This work provides a direct experimental result for the substrate-binding conformation of the product state of *E. cuniculi* FBPA.

## Abstract 2

The eukaryotic parasite *E. cuniculi* expresses a fructose bisphosphate aldolase that crystallizes readily in the presence of the partial substrate analog phosphate. This aldolase–phosphate structure and that of the sugar-bound Schiff base are reported. *E. cuniculi* aldolase displays a dimeric structure rather than the expected tetrameric quaternary structure.

## 1. Introduction

### 1.1. SSGCID

The Seattle Structural Genomics Center for Infectious Disease (SSGCID) is one of two consortia funded by NIAID to apply genome-scale approaches to the solution of protein structures from bio­defense organisms, as well as those causing emerging and re-emerging diseases. In its first three and a half years, the SSGCID has submitted >350 protein structures to the Protein Data Bank (PDB) and is on track to solve a further 100–125 per year going forward. For several organisms, this represents the majority of PDB submissions during this time, including 100% of the structures for *Ehrlichia*, *Anaplasma* and *Burkholderia*. SSGCID’s target-selection strategy has focused on drug targets, essential enzymes, virulence factors and vaccine candidates from a number of bacterial (*Bartonella*, *Brucella*, *Ehrlichia*, *Anaplasma*, *Rickettsia*, *Burkholderia*, *Borrelia* and *Mycobacterium*) and eukaryotic (*Babesia*, *Cryptosporidium*, *Toxoplasma*, *Giardia*, *Entamoeba*, *Coccidioides* and *Encephalitozoon*) pathogens, as well as ssDNA and negative-strand ssRNA viruses. More than 3000 targets have been selected to date, with >700 proteins being purified for crystallization trials. Crystallization screening and analysis of X-ray diffraction data sets for structure solution was performed at Emerald BioStructures.

Aldolases (EC 4.1.2) are enzymes that cleave aldols and they have been the subject of crystallographic study since 1971 (Heidner *et al.*, 1971 ▶), with a 4 Å resolution crystal structure being determined in 1984 (Carrell *et al.*, 1984 ▶). To date, more than 70 unique aldolase structures have been deposited in the PDB. The glycolytic enzyme fructose bisphosphate aldolase (EC 4.1.2.13) catalyzes the aldol cleavage of fructose 1,6-bisphosphate to dihydroxyacetone phosphate and glyceraldehyde 3-phosphate. It also catalyzes the reverse reaction. The hallmark of a class I aldolase is the presence of an active-site lysine residue which forms a Schiff base with the substrate. *Encephalitozoon cuniculi* is a microsporidian parasite that can infect a wide range of hosts, including rats and mice, and is one of 14 microsporidians known to be human pathogens. Here, we present two crystal structures of the class I fructose bisphosphate aldolase enzyme from *E. cuniculi*: one with the reactant bound at the active site and the other with the partial substrate mimic phosphate.

## 2. Materials and methods

All expression clones, purified proteins and protein structures produced by SSGCID are available to the scientific community.

### 2.1. Protein purification and crystallization

#### 2.1.1. Purification of *E. cuniculi* aldolase

Fructose bisphosphate aldolase (FBPA) from *E. cuniculi* (NCBI 6035; UniProt Q8SSM8; Pfam ID PF00274; EC 4.1.2.13) spanning the full-length protein from residues 1 to 338 was cloned into a pAVA0421 vector using ligation-independent cloning (LIC; Aslanidis & de Jong, 1990 ▶). The protein was expressed in *Escherichia coli* using BL21(DE3)R3 Rosetta cells and autoinduction medium in a LEX bioreactor. The frozen cells were resuspended in lysis buffer (25 m*M* HEPES pH 7.0, 500 m*M* NaCl, 5% glycerol, 30 m*M* imidazole, 0.025% sodium azide, 0.5% CHAPS, 10 m*M* MgCl_2_, 1 m*M* TCEP, 250 ng ml^−1^ AEBSF and 0.05 µg ml^−1^ lysozyme). The resuspended cell pellet was disrupted on ice for 30 min with a Virtis sonicator (408912; set at 100 W power, with alternating cycles of 15 s pulse-on and 15 s pulse-off). The cell debris was incubated with 20 µl Benzonase nuclease (25 units ml^−1^) at room temperature for 45 min and clarified by centrifugation on a Sorvall SLA-1500 at 14 000 rev min^−1^ for 75 min at 277 K. The protein was purified from the clarified cell lysate by immobilized metal-affinity chromatography on a HisTrap FF 5 ml column (GE Healthcare) equilibrated with binding buffer (25 m*M* HEPES pH 7.0, 500 m*M* NaCl, 5% glycerol, 30 m*M* imidazole, 0.025% sodium azide, 1 m*M* TCEP). The recombinant protein was eluted with 250 m*M* imidazole. The purification tag (MAHHHHHHMGTLEAQTQG­PGS) was not cleaved. This sample was further polished using a HiLoad 26/60 Superdex 75 size-exclusion chromatography (SEC) column (GE Healthcare). Pure fractions collected in SEC buffer (25 m*M* HEPES pH 7.0, 500 m*M* NaCl, 2 m*M* DTT, 0.025% sodium azide, 5% glycerol) in the major peak of the chromatogram (estimated molecular weight from SEC of 51 kDa) were pooled. The expected molecular weight of the protein was 40 kDa, which was verified by SDS–PAGE analysis. The protein was concentrated to 25 mg ml^−1^ using Amicon Ultra 10K centrifugal filters (Millipore). The concentrated sample was flash-cooled in liquid nitrogen and stored at 193 K prior to crystallization.

#### 2.1.2. Crystallization of *E. cuniculi* aldolase with the partial substrate phosphate

*E. cuniculi* aldolase at 25 mg ml^−1^ (0.6 m*M*) in SEC buffer (see above) was initially crystallized in PACT screen (Newman *et al.*, 2005 ▶) condition F10 and optimized with the ADDit Additive Screen (Emerald BioSystems). Aldolase stock solutions (0.4 µl) were mixed with reservoir (0.4 µl) and equilibrated against a 80 µl reservoir of precipitant (crystallant) using 96-well Compact Jr plates from Emerald BioSystems. The final crystallization conditions were 90 m*M* Bis-Tris propane pH 6.5, 18% PEG 3350, 18 m*M* NaKHPO_4_, 10 m*M* urea, with protein at 25 mg ml^−1^. Crystals were obtained by sitting-drop vapor diffusion at 290 K. Cryoprotection used a solution consisting of 80% well solution and 20% glycerol.

#### 2.1.3. Crystallization of *E. cuniculi* aldolase with the native substrate fructose 1,6-bisphosphate

*E. cuniculi* aldolase was co­crystallized with fructose 1,6-bisphosphate (FBP) in a condition based on PACT screen condition F10 with the phosphate reagent omitted from and FBP added to the protein solution (consisting of aldolase in SEC buffer). The final crystallization conditions were 100 m*M* Bis-Tris propane pH 6.5, 20% PEG 3350, 10 m*M* FBP, with protein at 20 mg ml^−1^. Crystals were obtained by sitting-drop vapor diffusion at 290 K. A solution consisting of 80% reservoir solution and 20% glycerol was used for cryoprotection.

### 2.2. Data collection, processing, structure solution and refinement

All data were indexed, integrated and scaled with the *XDS* suite (Kabsch, 2010 ▶). Data-collection and processing statistics are presented in Table 1 ▶. Refinement and validation parameters are presented in Table 2 ▶.

#### 2.2.1. *E. cuniculi* aldolase with the partial substrate phosphate

Data were collected on ALS beamline 5.0.1 as part of the Collaborative Crystallography Project. The wavelength was 0.9774 Å and the detector was an ADSC Quantum 210. The crystal-to-detector distance was 186 mm. 250 frames were collected with a width of 1° in ϕ at 100 K.

The structure was solved by molecular replacement with *Phaser* (McCoy *et al.*, 2007 ▶) using a search model prepared from human aldolase (PDB entry 1qo5; Dalby *et al.*, 2001 ▶) with the *CCP*4*i* (Winn *et al.*, 2011 ▶) interface to *CHAINSAW* (Stein, 2008 ▶). The structure was rebuilt in *Coot* (Emsley & Cowtan, 2004 ▶) and refined with *REFMAC*5 (Murshudov *et al.*, 2011 ▶).

#### 2.2.2. *E. cuniculi* aldolase with the native substrate fructose 1,6-­bisphosphate

Data were collected on SSRL beamline 7.1 at 100 K *via* a remote data-collection protocol. 220 frames, each of 0.5° in ϕ, were collected at a crystal-to-detector distance of 280 mm at an X-ray wavelength of 0.98 Å using an ADSC Q315 detector. The structure was solved by difference Fourier methods using the structure of *E. cuniculi* aldolase as determined for the phosphate-bound form, but with the phosphate ion and all water molecules removed from the input file.

## 3. Results and discussion

### 3.1. Overall structure

Fructose bisphosphate aldolase from *E. cuniculi* (EcFBPA) adopts the TIM-barrel fold typical of aldolases. It has 37–42% sequence identity to aldolases from other eukaryotes, such as those from *Drosophila*, *Plasmodium*, *Babesia* and mammalian aldolases B and C. The final phosphate-bound model contained one copy of FBPA spanning residues 2–337, one phosphate ion, one chloride ion and 192 water molecules. For FBP-bound FBPA, the final model contained one copy of FBPA spanning residues 2–338, one linear FBP molecule and 95 water molecules.

**Figure 1:**
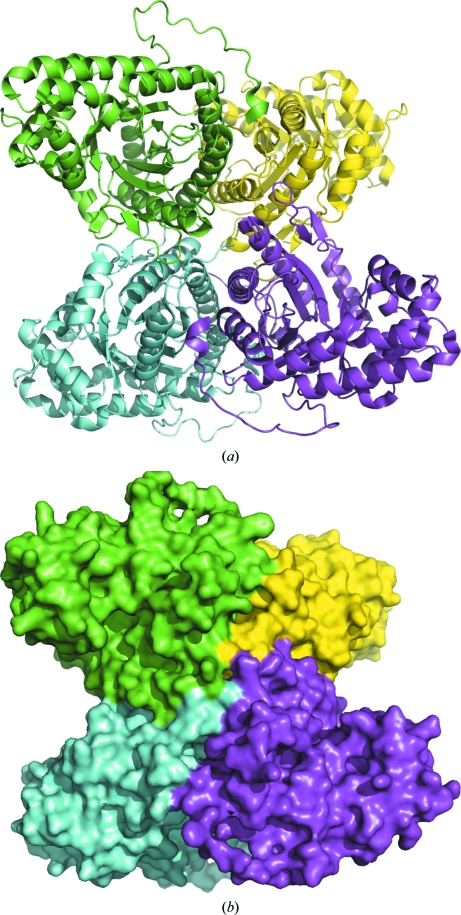
The typical tetrameric quaternary structure of FBPA is shown for rabbit muscle FBPA (PDB entry 1zai) as (*a*) cartoon and (*b*) surface plots.

Human and rabbit liver, muscle and brain aldolases adopt tetrameric quaternary structures, as do *Drosophila*, *Plasmodium*, *Babesia bovis*, *Leishmania mexicana* (PDB entries 1epx and 2qdg; Chudzik *et al.*, 2000 ▶; Lafrance-Vanasse & Sygusch, 2007 ▶), *Trypanosoma brucei* (PDB entry 1f2j; Chudzik *et al.*, 2000 ▶) and *Bartonella henselae* aldolases (PDB entry 3mmt; Gardberg *et al.*, 2011 ▶), although there is a D128V variant of rabbit muscle aldolase that forms a dimer (PDB entry 3bv4; Sherawat *et al.*, 2008 ▶) and *Giardia* aldolase (PDB entry 3gak; Galkin *et al.*, 2009 ▶) is dimeric. Evidence that the monomeric and dimeric forms of EcFBPA interconvert was found in the size-exclusion chromatogram, which contains a small peak for the dimer and a larger peak for the monomer. Dimers are found in both of the EcFBPA structures presented here; the computed buried surface area at the dimer interface is ∼3000 Å^2^ for both structures, while that for the tetramer interface of rabbit muscle FBPA A (PDB entry 1zai; St-­Jean *et al.*, 2005 ▶) is ∼13 500 Å^2^, with each dimer interface burying ∼4000 Å^2^ (Krissinel & Henrick, 2007 ▶). Figs. 1 ▶ and 2 ▶ show how the typical aldolase tetrameric quaternary structure has been disrupted here. The origin of the unusual dimeric quaternary structure of EcFBPA is not clear, but the shortened loop from Leu106 to Ile115 (Gly112–Thr112) suggests one possibility.

The final model for each structure showed good geometry (Table 2 ▶) as determined using the program *MolProbity* (Davis *et al.*, 2007 ▶).

### 3.2. Reactant state

**Figure 3:**
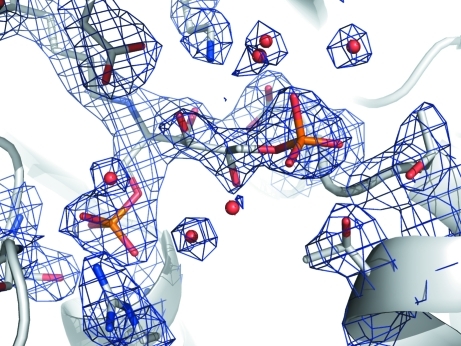
Weighted 2*F*_o_ − *F*_c_ electron-density map at 1.5σ for FBP and nearby residues in the active site of fructose bisphosphate aldolase from *E. cuniculi*. There is clear electron density for a Schiff base formed by Lys221 and the FBP molecule.

**Figure 4:**
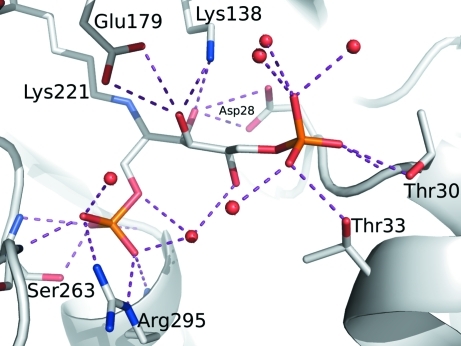
Binding environment for FBP bound at the active site of fructose bisphosphate aldolase from *E. cuniculi*.

**Figure 5:**
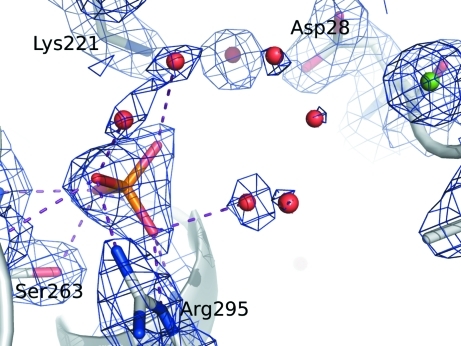
Weighted 2*F*_o_ − *F*_c_ electron-density map at 1.5σ for the phosphate ion and nearby residues in the active site of fructose bisphosphate aldolase from *E. cuniculi*.

As part of glycolysis, FBPA catalyzes the cleavage of fructose 1,6-­bisphosphate (FBP) to give glyceraldehyde 3-phosphate and dihydroxyacetone phosphate (DHAP). Using this protein sample, we solved a 2.37 Å resolution crystal structure of *E. cuniculi* FBPA bound to FBP (Tables 1 ▶ and 2 ▶). This structure has clear electron density for FBP bound at the active site (Fig. 3 ▶). There is a covalent bond between C atom C2 of the linear FBP molecule and the NZ atom of Lys221 of the protein. Furthermore, phosphate 1 of FBP makes hydrogen bonds to four amide N atoms as well as to the side chains of Arg295 and Ser263 (Fig. 4 ▶). The hydroxy groups along the linear carbon backbone of FBP also make hydrogen bonds to the protein, as well as to nearby water molecules. Finally, phosphate group 2 makes hydrogen bonds to side chains and to nearby water molecules. Similarly, there is clear electron density for the phosphate group in the 2.00 Å phosphate-bound structure (Fig. 5 ▶).

SSGCID’s interest in *E. cuniculi* FBPA is as a potential drug target, so it is illuminating to compare the active site of *E. cuniculi* FBPA with those of homologous mammalian enzymes. The orientation of the FBP molecule in the active site differs from that observed in the structure of human muscle aldolase (PDB entry 4ald; Dalby *et al.*, 1999 ▶; r.m.s.d. of non-H atoms in the FBP ligand of 2.23 Å; r.m.s.d. for C^α^ atoms over the whole protein monomer of 0.830 Å). However, the complex observed in 4ald does not show full formation of the Schiff base, suggesting that 4ald is a ‘preliminary hydrogen-bonded Michaelis complex before the formation of the covalent complex’ (Dalby *et al.*, 1999 ▶). It is more illuminating to note that the FBP conformation in *E. cuniculi* FBPA is essentially identical to that of rabbit muscle aldolase A (PDB entry 1zai; r.m.s.d. of non-H atoms in the FBP ligand of 0.40 Å; r.m.s.d. for C^α^ atoms over the whole protein of 0.580 Å).

## 4. Conclusion

**Figure 6:**
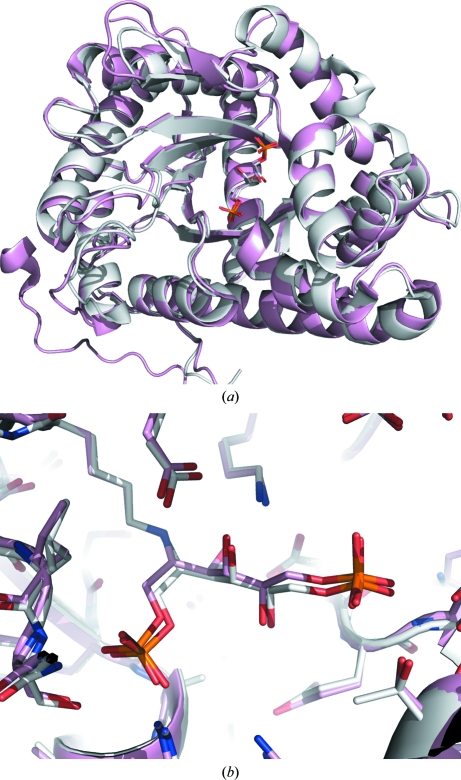
The (*a*) overall and (*b*) active-site structures of the reactant Schiff-base state are similar to those of some other aldolases in the reactant state (PDB entries 1zai, 3mmt and 2qdg), especially rabbit muscle FBPA A (PDB entry 1zai), which is shown here.

Crystal structures of fructose bisphosphate aldolase from *E. cuniculi* in complex with its reactant and with the partial substrate analog phosphate have been determined at 2.37 and 2.0 Å resolution, respectively. The structure of the reactant Schiff-base state is similar to those of some other aldolases in the reactant state (PDB entries 1zai, 3mmt and 2qdg), especially rabbit muscle FBPA A (PDB entry 1zai; Fig. 6 ▶). The similarity to the active site of mammalian FBPA A suggests that the design of a specific inhibitor for *E. cuniculi* FBPA (that does not inhibit the homologous human enzyme) would be exceptionally challenging and would have to rely on exploiting very minor differences in the chemical environment in and near the active site.

## Supplementary Material

PDB reference: fructose bisphosphate aldolase, phosphate-bound, 3mbd

PDB reference: FBP-bound, 3mbf

## Figures and Tables

**Figure 2:**
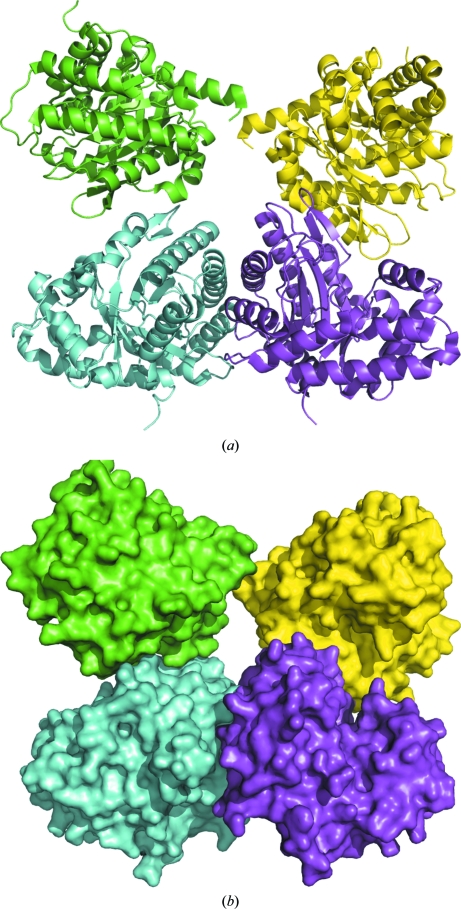
In *E. cuniculi* FBPA, the quaternary structure is dimeric. (*a*) Cartoon and (*b*) surface plots are shown for the phosphate-bound structure.

**Table 1:** Data-collection statistics Values in parentheses are for the highest of 20 resolution shells.

	Phosphate-bound	FBP-bound
Space group	*C*222_1_	*C*222_1_
Unit-cell parameters (Å)	*a* = 121.960, *b* = 137.610, *c* = 62.230	*a* = 121.460, *b* = 135.820, *c* = 61.540
Resolution range (Å)	35.34–2.0 (2.05–2.00)	28.03–2.37 (2.43–2.37)
Unique reflections	35720	21046
Multiplicity	6.2 (6.1)	4.4 (4.4)
Completeness (%)	99.8 (99.3)	99.7 (99.6)
*R*_merge_†	0.056 (0.489)	0.081 (0.479)
Mean *I*/σ(*I*)	21.830	15.350

† *R*_merge_ = 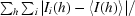 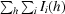

**Table 2:** Refinement and validation statistics

	Phosphate-bound	FBP-bound
Resolution range (Å)	35.34–2.00	28.07–2.37
*R*_cryst_†	0.162	0.165
*R*_free_†	0.190	0.208
R.m.s.d. bonds (Å)	0.015	0.016
R.m.s.d. angles (°)	1.395	1.499
Protein atoms	2631	2617
Nonprotein atoms	198	114
Mean *B* factor (Å^2^)	29.4	24.9
Ligand *B* factor (Å^2^)	37.8	26.1
Residues in favored region (%)	97.9	98.51
Residues in allowed region (%)	1.04	1.49
*MolProbity* score [percentile]	1.13 [100th]	1.32 [100th]
PDB code	3mbd	3mbf

† *R*_cryst_ = 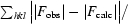 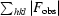. *R*_free_ was calculated using the 5% of the reflections that were omitted from the refinement.
